# Glucocorticoid Receptor (GR) antagonism as disease-modifying treatment for MDD with childhood trauma: protocol of the RESET-medication randomized controlled trial

**DOI:** 10.1186/s12888-023-04830-9

**Published:** 2023-05-11

**Authors:** F. Linsen, C. Broeder, M. S. C. Sep, J. E. Verhoeven, P. M. Bet, B. W. J. H. Penninx, O. C. Meijer, C. H. Vinkers

**Affiliations:** 1 Department of Psychiatry, Amsterdam University Medical Center Location Vrije Universiteit Amsterdam, Amsterdam, 1081 HV The Netherlands; 2 Department of Anatomy & Neurosciences, Amsterdam University Medical Center, Vrije Universiteit Amsterdam, Amsterdam, 1081 HV The Netherlands; 3 GGZ inGeest Mental Health Care, Amsterdam, 1081 HJ The Netherlands; 4 Department of Clinical Pharmacology and Pharmacy, Amsterdam UMC, Vrije Universiteit Amsterdam, Amsterdam, 1081 HV the Netherlands; 5 Amsterdam Public Health, Mental Health Program and Amsterdam Neuroscience, Mood, Anxiety, Psychosis, Sleep & Stress Program, Amsterdam, The Netherlands; 6 Division of Endocrinology, Department of Internal Medicine, Leiden University Medical Center, Leiden University, Leiden, 2333 ZA the Netherlands; 7 Einthoven Laboratory for Experimental Vascular Medicine, Leiden University Medical Center, Leiden University, Leiden, 2333 ZA the Netherlands

**Keywords:** Major depressive disorder, Childhood trauma, Glucocorticoid receptor, Mifepristone, Randomized controlled trial

## Abstract

**Background:**

Major depressive disorder (MDD) is a heterogeneous psychiatric disorder. Childhood trauma (CT, emotional/physical/sexual abuse or neglect before the age of 18) is one of the largest and most consistent risk factors for development and poor course of MDD. Overactivity of the HPA-axis and the stress hormone cortisol is thought to play a role in the vulnerability for MDD following exposure to CT. Rodent experiments showed that antagonism of the glucocorticoid receptor (GR) at adult age reversed the effects of early life stress. Similarly, we aim to target MDD in individuals with CT exposure using the GR antagonist mifepristone.

**Methods:**

The RESET-medication study is a placebo-controlled double-blind randomized controlled trial (RCT) which aims to include 158 adults with MDD and CT. Participants will be randomized (1:1) to a 7-day treatment arm of mifepristone (1200 mg/day) or a control arm (placebo). Participants are allowed to receive usual care for MDD including antidepressants. Measurements include three face-to-face meetings at baseline (T0), day 8 (T1), week 6 (T2), and two online follow-up meetings at 12 weeks (T3) and 6 months (T4). A subgroup of participants (*N* = 80) are included in a fMRI sub-study (T0, T2). The main study outcome will be depressive symptom severity as measured with the Inventory of Depressive Symptomatology—Self Rated (IDS-SR) at T2. Secondary outcomes include, among others, depressive symptom severity at other time points, disability, anxiety, sleep and subjective stress. To address underlying mechanisms mifepristone plasma levels, cortisol, inflammation, epigenetic regulation and fMRI measurements are obtained.

**Discussion:**

The RESET-medication study will provide clinical evidence whether GR antagonism is a disease-modifying treatment for MDD in individuals exposed to CT. If effective, this hypothesis-driven approach may extend to other psychiatric disorders where CT plays an important role.

**Trial registration:**

The trial protocol has been registered 01–02-2022 on ClinicalTrials.gov with ID “NCT05217758”.

**Supplementary Information:**

The online version contains supplementary material available at 10.1186/s12888-023-04830-9.

## Background

Major depressive disorder (MDD) is a recurrent and progressive psychiatric disorder [1, 2], and a leading cause of disability [3]. Around 10–30% of patients do not benefit from evidence-based treatments such as antidepressants and psychotherapy [4]. It is increasingly clear that MDD is a heterogeneous disorder with varying clinical phenotypes [5, 6]. Accordingly, novel therapeutic strategies for MDD may target specific subtypes of MDD, such as immunometabolic depression [7]. Childhood trauma (CT, emotional/physical/sexual abuse or neglect before the age of 18) is one of the most consistent risk factors for both developing MDD and a poorer course of disease [8, 9]. Estimates of CT prevalence in MDD vary, but it is thought that between 25 to 75% of MDD patients have experienced moderate to severe CT [10]. MDD following exposure to CT typically emerges earlier in life with more severe and chronic symptoms [11–13], more anxiety, suicidality, and insomnia, and reduced daily functioning [14, 15]. There is an unmet need for treatments that target the high burden of MDD with CT exposure.

CT is thought to activate the hypothalamic–pituitary–adrenal (HPA) axis and elevate cortisol in a sensitive developmental period. The effects of cortisol are likely mediated by binding to glucocorticoid receptors (GR) that are widely distributed in the brain. These processes early in life may result in long-lasting maladaptive effects on stress vulnerability and result in overall poorer (mental) health including an increased MDD risk [16]. There is compelling evidence for the changes in brain function and behaviour that may underlie disease vulnerability. These include structural and functional changes in brain networks [17, 18], working memory [19–21], emotional regulation [22–24], HPA-axis functionality [16, 25], and increased inflammation [26], but also psychological and lifestyle mechanisms [27].

In line with the notion of an overactivated HPA-axis during, and possibly after stress in early life, treatment with a GR antagonist emerges as a promising strategy from the animal literature [28–31]. In several studies, administration of the GR-antagonist mifepristone in adolescent rodents exposed to early life stress reversed increased freezing behaviour at adult age [28] and normalized open-field behaviour and corticosterone levels when administered at adult age [31]. Mifepristone also reversed changes in social behaviour in animals stressed during adolescence [30]. Thus, in rodents, the GR antagonist mifepristone seems to be able to counteract long-term GR overactivation following early life stress. In humans, such effects of GR antagonism have not been examined. Nevertheless, the GR antagonist mifepristone has been widely used for decades to treat Cushing’s syndrome [32]. Based on beneficial effects on psychiatric symptoms observed in Cushing’s disease, mifepristone has also been investigated for psychotic depression (7-day mifepristone regimen 300–1200 mg/day) [33–38]. A combined analysis of five RCTs showed an overall significant effect of mifepristone (*N* = 793) vs. placebo (*N* = 595) in reducing psychotic symptoms in psychotic depression, but significantly more so in patients with high plasma mifepristone levels after 7 days (d = 0.3) compared to low plasma levels (d = 0.05) [39]. From these studies, a 7-day mifepristone treatment is known to be generally well-tolerated and adverse events (AEs) were not significantly different compared to placebo treatment.

The REStoring mood after Early life Trauma (RESET) study is a double-blind, placebo-controlled, randomized trial that tests the hypothesis whether a 7-day treatment using the GR antagonist mifepristone (1200 mg) improves depression in patients with moderate to severe MDD and CT.

## Methods

### Study design

**Fig. 1:**
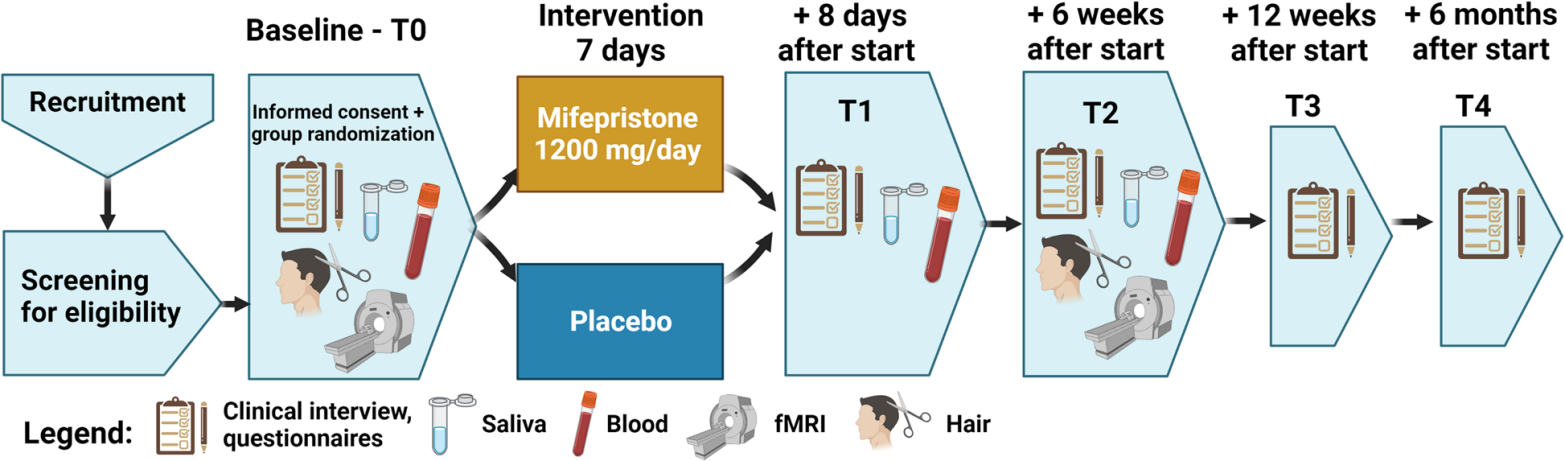
Flowchart of the RESET-medication time-points and measurements. Created with BioRender.com

This is a mono-center, placebo-controlled, double-blind randomized controlled trial (RCT) testing the efficacy of a 7-day treatment of mifepristone (1200 mg/day) or placebo in *N* = 158 patients with MDD and CT. The study consists of 5 measurement time-points: baseline (T0), post-intervention at 1 week (T1), 6 weeks (T2) after the start of the intervention, and two online follow-up measurements (T3 at 12 weeks, T4 at 6 months) (see Fig. 1). The optional (f)MRI sub-study is conducted at separate visits around the T0 and T2 time points. Throughout the study, participants are allowed to continue or start their treatment as usual (TAU) in parallel to the RCT. The RESET-medication protocol is reported according the SPIRIT guidelines and methodology [40] (see Additional file 1).

### Recruitment and study settings

Recruitment of participants occurs through a study website (www.jeugdtrauma-depressie.nl), where information can be found about the RCT and how to participate. The study website is advertised on websites of the Dutch patient federation for depression ‘Depressievereniging.nl’, the subsidy party ‘Hersenstichting.nl’, and (social) media. Also, at several mental health clinics throughout the Netherlands therapists treating patients with MDD and CT inform patients about the RESET study.

The study website screens for suitable participants based on depressive symptoms (Inventory of Depression Severity – Self Rated; IDS-SR) and CT (short form of the Childhood Trauma Questionnaire; CTQ-SF). Eligible participants are contacted by research assistants (RAs) within 2 weeks via telephone for further screening. If the participant is deemed suitable for participation, the information letter is sent out to consider all relevant information regarding participation. Once participants agree to participate, T0, T1, T2 visits are conducted at the research center of Amsterdam UMC, location VUmc. Follow-up measurements at T3, T4 are conducted via video calling and online questionnaires. The optional fMRI sub-study is conducted at the Spinoza center for neuroimaging in Amsterdam, the Netherlands. All measurements are performed by certified RAs. Participants receive €20 for each RCT study time-point (T0-T5), and €30 for each fMRI visit (T1, T2).

### Eligibility criteria

Inclusion criteria are: 1) Age of ≥ 18 years and able to give written informed consent (IC); 2) current moderate to severe depression with a score of ≥ 26 on the IDS-SR; 3) DSM-5 diagnosis of MDD confirmed with clinical interview (Mini International Neuropsychiatric Interview—Simplified; MINI-S) during T0; 4) Moderate to severe CT before the age of 18 with a score above a validated cut-off for moderate to severe CT on one or more of the following domains using the CTQ-SF: physical neglect: score ≥ 10, emotional neglect: score ≥ 15, sexual abuse: score ≥ 8, physical abuse: score ≥ 10; emotional abuse: score ≥ 13; 5) Women of child bearing potential (WOCBP) agree to use a non-hormonal contraceptive method (e.g. condom) during the intervention period and up to 1 month after the intervention based on the abortive effects of mifepristone via progesterone receptor antagonism.

Exclusion criteria are primarily checked by self-report during the telephone screening and consist of: 1) primary diagnosis of post-traumatic stress disorder (PTSD) or Acute Stress Disorder (ASD) where a diagnosis must have been received from a physician/mental health practitioner; 2) Lifetime diagnosis of borderline personality disorder (BPD), bipolar disorder, schizophrenia or current substance abuse where a diagnosis must have been received from a physician/mental health practitioner; 3) Start of depression treatment (e.g. psychotherapy, antidepressants) in the week before or after the start of the intervention. Hereby, study participation does not intervene with patient needs of treatment; 4) Chronic adrenal insufficiency, diagnosed by a physician; 5) Female participants that have a history of unexplained vaginal bleeding or endometrial changes. 6) Female participants that are pregnant or breastfeeding. Pregnancy is excluded using a negative pregnancy test during the baseline visit; 7) Current use of drugs being CYP3A4 inhibitors/inductors/substrates, CYP2C8/9 substrates, glucocorticoid antagonists and systemic corticosteroids. A list of exclusion drugs is used for screening to prevent interactions with mifepristone. See Table 1 for an overview of the in- and exclusion criteria.

**Table 1:** Overview of inclusion and exclusion criteria

**Inclusion criteria**	**Exclusion criteria**
1. Age of ≥ 18 years	1. Primary PTSD or ASD
2. Moderate/severe MDD	2. Lifetime BPD, bipolar disorder, schizophrenia or current substance abuse
3. Moderate/severe CT	3. Start of depression treatment in the week before or after the start of the intervention
4. For WOCBP, use of a non-hormonal contraceptive method (e.g. condom) during the intervention period and up to 1 month after the intervention	4. Chronic adrenal insufficiency
	5. A history of unexplained vaginal bleeding or endometrial changes.
	6. Pregnancy or breastfeeding
	7. Current drug use of CYP3A4 inhibitors/inductors/substrates, CYP2C8/9 substrates, glucocorticoid antagonists and systemic corticosteroids

### Sample size

Based on preclinical evidence [28–31] and our targeted treatment of MDD with moderate to severe CT in RESET, we hypothesize a larger effect size compared to clinical trials of psychotic depression with an effect size of d = 0.3 [39]. Therefore, it is estimated that this targeted disease-modifying treatment in a clinically specific group may have a robust treatment effect on the primary outcome measure with a medium effect size (Cohen’s d = 0.50, α = 0.05, two-tailed, power 80%). This requires 63 participants per group. Previous randomized studies in psychotic depression using a 7-day mifepristone treatment (300–1200 mg/day, *N* = 1388) found average dropout rates of around 20% [39]. Therefore, a total sample size of *N* = 158 is required, with *N* = 79 in each treatment arm of mifepristone and placebo.

For the (f)MRI sub-study, 40 participants will be included per treatment arm (total of *N* = 80). This required sample size is calculated for our primary fMRI outcome measure: dynamical resting-state connectivity in response to a Trier Social Stress Test (TSST) [41]. Here we specifically aim to detect the influence of mifepristone vs. placebo on variation in network connectivity between the acute and recovery phase of this stress response. Currently, the effect size of mifepristone on these dynamics is unknown. However, we do know that the difference in resting-state network connectivity between the acute and delayed response phase is medium in healthy individuals (Cohen's f = 0.315) [42]. We also know from work in rodents that mifepristone has a medium effect on the structural integrity of the brain (Cohen's f = 0.316) [43]. Therefore, we want to be able to detect a medium effect size in our fMRI sub study. Calculations using R-package WebPower [44] revealed that we need two equal groups of 40 individuals to detect a medium effect (Cohen's f = 0.32) in within-between subject interactions in a repeated-measures ANOVA with 2 groups (i.e. placebo and mifepristone) and 2 measures (i.e. acute and delayed phase), and obtain a power of 80 percent with type I error rate of 0.05.

### Study procedures

#### Informed consent (IC) and baseline assessment

Participants must personally sign and date the latest approved version of the IC form before any study-specific procedures are performed. The IC will be signed at the beginning of the baseline visit (T0) at the research center. Written versions of the IC will be presented to the participants, where it is clearly stated that the participant is free to withdraw from the study at any time for any reason, and with no obligation to give the reason for withdrawal. After the IC is signed by the participant, the treating therapist or physician (TAU) is informed by the researcher regarding the RCT participation. In case of additional participation in the (f)MRI sub-study, there will be a separate IC and safety form (contraindications MRI) to be signed by the participant before the baseline scanning session is initiated.

#### Randomization and blinding

Once the IC is signed during the baseline visit (T0), randomization is performed by the RA carrying out the measurement. A web-based randomization program using a list of unique randomization codes with block randomization (ratio 1:1, mifepristone *N* = 79, placebo *N* = 79) is used. Randomization will be stratified for fMRI participation. This way, the fMRI subgroups are well balanced with mifepristone and placebo interventions. During the T0 visit, the randomization code and study medication prescription are sent to the hospital pharmacy for dispensing. The randomization codes are kept by the hospital pharmacy and the data management team of Amsterdam UMC, location VUmc, out of reach from the involved researchers until data collection is completed. If de-blinding of study medication is needed in case of Serious Adverse Events (SAEs) for urgent medical reasons, the pharmacy can be contacted by the researchers.

#### Intervention

Participants will receive either mifepristone (1200 mg/day) or placebo for 7 consecutive days. Tablets are packaged in HDPE-bottles containing 28 tablets of 300 mg each or placebo. Placebo tablets are matched to mifepristone tablets in shape, smell and colour. Study medication is orally ingested in the morning during a meal, 4 tablets of mifepristone (total 1200 mg) or placebo. Study adherence is assessed by drug tablet return and mifepristone plasma levels at T1.

### Assessments, outcomes and instruments

#### Primary outcome

The primary outcome is depressive symptom severity as measured by the IDS-SR 6 weeks after the start of the intervention (T2). The IDS-SR is a questionnaire with high internal consistency and good concurrent validity [45].

#### Secondary outcomes

- MDD remission (< 14 on IDS-SR, confirmed with the MINI-S), 6 weeks (T2) after the start of the intervention.
- Short-term depressive symptom severity (IDS-SR) and MDD remission (MINI-S), 1 week (T1) after the start of the intervention.
- Long-term depressive symptom severity (IDS-SR) and MDD remission (MINI-S), 12 weeks (T3) and 6 months (T4) after the start of the intervention.
- Treatment response (50% decrease in IDS-SR score) at all post-intervention time-points
- Disability (WHO Disability Schedule 2.0; WHO-DAS II) [46], at all post-intervention time-points.
- Sleep (Pittsburgh Sleep Quality Index; PSQI) [47], at all post-intervention time-points.
- Subjective stress (Perceived Stress Scale; PSS) [48], at all post-intervention time-points.
- Anxiety symptoms (Beck Anxiety Inventory; BAI) [49], at all post-intervention time-points.

### Descriptive variables

The following descriptive variables and potential moderators/mediators will be assessed in order to thoroughly describe the study sample.

- Age, sex, smoking, BMI, physical activity (International Physical Activity Questionnaire; IPAQ) [50], alcohol use (Alcohol Use Disorders Identification Test; AUDIT) [51].
- Current treatment (type, duration and frequency; self-composed questionnaire TAU)
- Adverse side effects of study medication (self-composed questionnaire)
- Characteristics (e.g. type, chronicity) of CT (CTQ-SF and Maltreatment and Abuse Chronology of Exposure; MACE) [52]
- Resilience (Connor Davidson Resilience Scale; CD-RISC2) [53]
- Coping style (COPE-16) [54]
- Personality traits (NEO Five-Factor Inventory; NEO-FFI) [55]
- Comorbid PTSD/ASD diagnosis (non-primary diagnosis) (MINI-S) [56]
- Life events (List of Threatening Experiences; LTE) [57]
- Social support (Social Support List; SSL) [58]
- Suicidal ideation and behaviour (Columbia-Suicide Severity Rating Scale; C-SSRS) [59].This is used within a suicide prevention protocol in case participants show a risk regarding suicidality when they answer positively on the suicidality item of the MINI-S, which is assessed at every study time-point.

### Biological markers

Various stress related biomarkers are assessed pre- and post-intervention to better understand the underlying mechanism of a clinical response to mifepristone treatment (see Table 2):

- Saliva samples are taken at home pre- and post-intervention (T0, T1, T2) to assess the Cortisol Awakening Response (CAR), directly at awakening, at 30 min, and 45 min after awakening [60]. Also, one evening sample between 21-22 pm is collected, as ultradian rhythmicity shows lowest variability in the evening, resulting in reliable cortisol assessments [61].
- Hair samples are collected pre- and post-intervention (T0, T2) to retrospectively assess cumulative cortisol levels, as it is a good reflection of a more chronic cortisol exposure [62].
- Blood samples are collected pre- and post-intervention (T0, T1, T2) for various biomarkers:
  - ◦ Mifepristone blood plasma levels are assessed from T1 blood samples, 1 day after the last study medication. Based on previous clinical trials with a 7-day mifepristone treatment, high plasma levels (HPL; ≥ 1637 ng/mL) are expected to be associated with a higher clinical response compared to low plasma levels (< 1637 ng/mL). Due to inter-individual variability in mifepristone metabolization, 65% of participants are expected to reach HPL with a 1200 mg daily dose [39].
  - ◦ Inflammatory markers like C-reactive protein (CRP), Tumor Necrosis Factor-alpha (TNF-α) and Interleukin-6 (IL-6) are assessed as these have been found to be elevated in patients with MDD or CT [63].
  - ◦ Epigenetic regulation is assessed from somatic white blood cells through genome-wide microarrays, as preclinical findings indicated mifepristone could alter DNA expression and thereby reverse the negative effects of long term GR overactivation [64].

**Table 2:** Overview of RESET-medication measurements per study time-point

**Construct**	**Instrument**	**Method**	**Screening**	**T0**	**T1**	**T2**	**T3**	**T4**
Depression symptom severity	IDS-SR	SR	X	X	X	X	X	X
Childhood trauma	CTQ-SF	SR	X					
MDD diagnosis	MINI-S	Int		X	X	X	X	X
Suicidality	C-SSRS	Int		^b^	^b^	^b^	^b^	^b^
Anxiety symptoms	BAI	SR		X	X	X	X	X
Disability	WHODAS-II	SR		X	X	X	X	X
Sleep duration	ISI	SR		X	X	X	X	X
Perceived stress	PSS	SR		X	X	X	X	X
Stressful life events	LTE	SR		X		X	X	X
Social support	SSL	SR		X	X	X	X	X
Medication adherence-TAU	MARS-5	SR		X	X	X	X	X
Medication adherence-RCT	Pill count	Int			X			
Adverse side effects	SC	SR			X^c^			
Demographics	SC	Int		X				
Past/current smoking	SC	SR		X				
Physical activity	IPAQ	SR		X				
Alcohol use	AUDIT	SR		X				
Drug use	SC	SR		X				
PTSD symptoms	MINI-S	Int		X				
Current/previous TAU	SC	Int		X				X
Resilience	CD-RISC2	SR		X				
CT characteristics	MACE	SR		X				
Coping style	COPE-16	SR		X				
Personality traits	NEO-FFI	SR		X				
Contraindications mifepristone	SC	Int	X	X				
Pregnancy test	Urine	BM		X				
Cortisol	Hair Saliva	BM BM		X X	X	X X		
Inflammation, epigenetics, mifepristone plasma levels	Blood	BM		X	X	X		
fMRI sub-study^a^: Stress-related biological measures	Structural MRI, resting-state fMRI task-based fMRI; Saliva Heart-rate	BM BM BM BM BM		X X X X X		X X X X X		
fMRI sub-study: Subjective stress measures	PANAS STAI-S VAS DARS	SR SR SR SR		X X X X		X X X X		

*T0* Baseline, *T1* 1 week after start study medication, *T2* 6 weeks after start study medication, *T3* 12 weeks after start study medication, *T4* 6 months after start study medication, *TAU* treatment as usual, *SR* Self-Report, *Int* Interview by researcher, *BM* Biological measure, *IDS-SR* Inventory of depressive symptomatology-self rated, *MINI-S* Mini International Neuropsychiatric Interview-Simplified, *CTQ-SF* Childhood Trauma Questionnaire – Short Form, *C-SSRS* Columbia-Suicide Severity Rating Scale, *BAI* Beck Anxiety Inventory, *WHODAS-II* WHO Disability Schedule-II, *ISI* Insomnia Severity Scale, *PSS* Perceived Stress Scale, *LTE* List of Threatening Experiences, *SSL* Social Support List, *MARS-5* Medication Adherence Report Scale, *SC* self-composed questionnaire, *IPAQ* International Physical Activity Questionnaire, *AUDIT* Alcohol Use Disorders Identification Test, *CD-RISC2* 2-item Connor-Davidson Resilience Scale, *MACE* Maltreatment and Abuse Chronology of Exposure questionnaire, *COPE-16* Coping Orientation to Problems Experienced*, NEO-FFI* NEO Five-Factor Inventory, *fMRI* functional Magnetic Resonance Imaging

^a^only in fMRI sub-group

^b^only assessed if patient shows risk regarding suicidality

^c^repeated 2 weeks after T1

### Neuro-imaging

In order to test for potential neurobiological effects of mifepristone treatment, a (f)MRI sub-study will be conducted in a sub-group of *N* = 80 patients (*N* = 40 per group), at baseline (T0) and repeated at post-intervention (T2). Within each visit (T0, T2) there are two (f)MRI sessions which will be conducted after the TSST in order to assess the acute (0–40 min) and recovery (60–100 min) phases of stress system dynamics in response to a psychosocial stressor. The scanning procedure consists of anatomical scans (T1, Diffusion Tensor Imaging; DTI), functional resting-state, and two commonly used fMRI tasks: 1) N-back working memory task [65] where participants are presented a series of stimuli one-by-one and are asked to decide for each stimulus whether it matches the one displayed n (1,2, 3, etc.) trials ago. 2) Situation-focused volitional reappraisal task [66] where participants are presented with positive, negative or neutral pictures and are asked to either positively reinterpret the displayed situation (situation-focused volitional reappraisal) or to attend the picture without manipulating the emotional response to it (control condition). After each picture, participants were asked to rate their emotional state using a Self-Assessment Manikin for valence (SAM) [67], which served as the outcome measure. Pictures are selected from the International Affective Picture System (IAPS) based on normative ratings in valence and arousal [68].

For all functional scans T2*-weighted echo planar images (EPIs), sensitive to blood oxygenation level-dependent (BOLD) contrast will be obtained, covering the entire brain. Stress system dynamics in response to the TSST will also be assessed on the level of endocrine responses (salivary cortisol and alpha-amylase), psychophysiology (heart rate) and behaviour (in fMRI tasks). All task stimuli are presented using the stimulus presenter software Presentation (Neurobehavioral Systems, Albany, CA). Scanning protocols are identical for baseline and post-intervention assessments and are conducted at a 3 Tesla scanner at the Spinoza Centre for neuroimaging, Amsterdam, the Netherlands.

### Statistical analyses

All randomized participants will be included in the analyses according to the intention-to-treat (ITT) principle. Primary and secondary outcomes will be assessed over time between groups using a linear mixed models (LMM) as it can account for missing data. The primary outcome is depressive symptom severity using the continuous outcome of the IDS-SR questionnaire completed 6 weeks after the start of the intervention (T2). Secondary outcomes are assessed throughout the study from 1 week (T1) to 6 months (T4) after the start of the intervention. All (primary and secondary) outcomes will also be stratified by low or high plasma mifepristone levels based on Block et al. 2018. The significance threshold is set at *p* = 0.05, with correction for multiple testing for secondary outcomes. Descriptive and biological factors will be examined as potential determinants of mifepristone’s treatment effects by studying moderation and mediation. Adverse side effects from the treatment are tested between groups with a 2-sample t-test. Data from the (f)MRI sub-sample will be pre-processed and analyzed using existing pipelines (fMRIPrep) and FSL (FMRIB Software Library) and is assessed in relation to depression severity and treatment condition.

### Data management and quality assurance

Data will be collected and stored digitally using an electronic Case Report From (eCRF) in Castor EDC (a certified online data collection tool for medical research) or Survalyzer (during screening), with the exception of the signed IC and biological samples. Study data from questionnaires and clinical interviews in Castor EDC, collected hair, saliva and blood samples are stored, handled, and de-identified using the ID number. Only the local research team, the pharmacy staff, the data managers at Amsterdam UMC, location VUmc and the study monitor will have access to Castor EDC and the separate database that connects the ID study number to a person. Screening information is used to report the progress of inclusion according the CONSORT criteria that is needed for publishing (‘not meeting inclusion criteria’, ‘declined to participate’ or ‘other reasons’). The Clinical Monitoring Center (CMC) from the Amsterdam UMC will ensure that the study is adequately monitored during and after completion of data-collection.

Data will be stored 15 years upon completion of the study. For the collection and use of blood, saliva and hair samples for the current study, consent is needed from the participant. Blood samples will be stored at the central VUmc biobank in cryovials at -80 °C, and are stored for 15 years if the participant provided explicit consent. If the participant does not give consent for the biobank, blood samples will be destroyed within 5 years after data collection is completed, together with the saliva and hair samples. Saliva samples will be stored at the central VUmc laboratory and hair samples will be stored centrally at the study site (Amsterdam UMC, location VUmc) at room temperature.

### Adverse event reporting

Adverse events (AEs) are defined as any undesirable experience occurring to a participant during the study, whether or not considered related to the investigational product or trial procedure. AEs are actively inquired during post-intervention visits. Additionally, a side-effects questionnaire is filled out by participants at T1 and is repeated 2 weeks later. These time points were chosen based on mifepristone's half-life of 85 h at steady-state with 600 mg/day. Participants, who experience severe side effects, are allowed to reduce the daily dose to 600 mg for the rest of the 7 day period, if deemed medically safe. All serious adverse event (SAE) related to (mental) health excesses or death are reported to the accredited Medical Research Ethical Committee (MREC) that approved the protocol according to its requirements.

### Trial status

The RESET-medication trial is pre-registered at ClinicalTrials.gov (registered at 01–02-2022, identification number: NCT05217758). Participants are currently being recruited and enrolled. Date of first enrolment was on 09–12-2021 and first enrolment in the fMRI sub-study was on 05–12-2022.

## Discussion

Based on preclinical and clinical work, the rationale for the RESET-medication randomized double-blind and placebo-controlled trial is to examine the possible beneficial 7-day treatment with the GR antagonist mifepristone (1200 mg per day) in patients with moderate to severe MDD and CT. In rodent studies, mifepristone’s reversal effects of early life stress at adult age at the behavioural and neuroendocrine level provide support for the RESET study [28, 31]. In clinical studies 7-day mifepristone treatment benefitted patients with psychotic depression where mifepristone plasma levels were sufficiently high [39]. The RESET-medication trial will assess whether targeting the GR in the large group of adult MDD patients that have been exposed to moderate to severe CT can alleviate depressive symptoms in this MDD subgroup. Moreover, this study will also shed further light on the biological mechanisms underlying a potential clinical response following targeted GR antagonism using neuro-imaging and various biological factors such as cortisol, inflammation and epigenetic regulation. The RESET-medication study may pave the way for more targeted treatments for MDD subtypes, in this case MDD with exposure to moderate to severe CT. This may not only help these patients for whom current evidence-based treatments are insufficient^13^, but also increase our understanding of the complex interplay between biological and psychological factors that shape the lifelong negative consequences of CT.

## Supplementary Information

**Additional file 1:** **SPIRIT 2013 Checklist.** SPIRIT 2013 Checklist: Recommended items to address in a clinical trial protocol and related documents.

## Data Availability

Individual participant-level data (IPD) that underlie the study results will be shared in scientific, peer-reviewed journals (text, tables, figures and appendices). Trial data can be requested by submitting an analysis plan and data request to the principle investigator (PI) Christiaan Vinkers who will check on the adequacy and relevance of the proposed data analyses. After approval, the data management team of the Amsterdam UMC, location VUmc will provide permission and access to use the data.

## Abbreviations

AE: Adverse event
ANOVA: Analysis Of Variance
ASD: Acute Stress Disorder
AUDIT: Alcohol Use Disorders Identification Test
BAI: Beck Anxiety Inventory
BMI: Body Mass Index
BOLD contrast: Blood Oxygenation Level-Dependent contrast
BPD: Borderline Personality Disorder
CAR: Cortisol Awakening Response
CD-RISC: Connor-Davidson Resilience Scale
CMC: Clinical Monitoring Center
COPE-16: Coping Orientation to Problems Experienced
CRA: Clinical Research Associate
CRP: C-Reactive Protein
C-SSRS: Columbia-Suicide Severity Rating Scale
CT: Childhood Trauma
CTQ-SF: Childhood Trauma Questionnaire—Short Form
DNA: Deoxyribonucleic acid
DSM-5: Fifth edition of the Diagnostic and Statistical Manual of Mental Disorders
DTI: Diffusion Tensor Imaging
eCRF: Electronic Case Report Form
EPIs: Echo Planar Images
fMRI: Functional Magnetic Resonance Imaging
FSL: FMRIB Software Library
GR: Glucocorticoid Receptor
HDPE: High-density polyethylene
HPA: Hypothalamic Pituitary Adrenal
HPL: High Plasma Level
IAPS: International Affective Picture System
IC: Informed Consent
IDS-SR: Inventory of Depressive Symptomatology—Self Rated
IL-6: Interleukin-6
IPAQ: International Physical Activity Questionnaire
ISI: Insomnia Severity Scale
ITT: Intention-To-Treat
LMM: Linear Mixed Models
LTE: List of Threatening Experiences
MACE: Maltreatment and Abuse Chronology of Exposure
MARS-5: Medication Adherence Report Scale
MDD: Major Depressive Disorder
MINI-S: Mini International Neuropsychiatric Interview – Simplified
MREC: Medical Research Ethical Committee
NEO-FFI: Neuroticism–Extroversion–Openness Five Factor Inventory
PI: Principal Investigator
PSS: Perceived Stress Scale
PSQI: Pittsburgh Sleep Quality Index
PTSD: Posttraumatic Stress Disorder
RA: Research Assistant
RCT: Randomized Controlled Trial
RESET: REStoring mood after Early life Trauma
SAE: Serious Adverse Event
SAM: Self-Assessment Manikin
SSL: Social Support List
TAU: Treatment As Usual
TNF-α: Tumor Necrosis Factor-alpha
TSST: Trier Social Stress Test
UMC: University Medical Centers
VUmc: VU University Medical Center
WHODAS: WHO Disability Schedule
WMO: Medical Research Involving Human Subjects Act
WOCBP: Woman Of Child Bearing Potential
